# Adaptive use of interaction torque during arm reaching movement from the optimal control viewpoint

**DOI:** 10.1038/srep38845

**Published:** 2016-12-12

**Authors:** Van Hoan Vu, Brice Isableu, Bastien Berret

**Affiliations:** 1 CIAMS, Univ. Paris-Sud., Université Paris-Saclay, Orsay, France; 2 CIAMS, Université d’Orléans, 45067, Orléans, France; 3 Aix Marseille Univ, PSYCLE, Aix-en-Provence, France

## Abstract

The study aimed at investigating the extent to which the brain adaptively exploits or compensates interaction torque (IT) during movement control in various velocity and load conditions. Participants performed arm pointing movements toward a horizontal plane without a prescribed reach endpoint at slow, neutral and rapid speeds and with/without load attached to the forearm. Experimental results indicated that IT overall contributed to net torque (NT) to assist the movement, and that such contribution increased with limb inertia and instructed speed and led to hand trajectory variations. We interpreted these results within the (inverse) optimal control framework, assuming that the empirical arm trajectories derive from the minimization of a certain, possibly composite, cost function. Results indicated that mixing kinematic, energetic and dynamic costs was necessary to replicate the participants’ adaptive behavior at both kinematic and dynamic levels. Furthermore, the larger contribution of IT to NT was associated with an overall decrease of the kinematic cost contribution and an increase of its dynamic/energetic counterparts. Altogether, these results suggest that the adaptive use of IT might be tightly linked to the optimization of a composite cost which implicitly favors more the kinematic or kinetic aspects of movement depending on load and speed.

Human movement control is a complex process partly due to the non-linearity induced by the pluri-articulated nature of the skeletal system. Motion in the body structure leads to the emergence of interaction torque (IT) between segments (e.g. action of the forearm onto the upper-arm) but also within a segment (e.g. among the 3 main degrees of freedom of the shoulder joint for the upper-arm in 3D). Such coupled nonlinear dynamics implies that motion at one degree of freedom (DoF) may induce significant motion at the other ones. This property requires the central nervous system (CNS) to rely on effective computational and neural principles for accurately controlling movement even for simple daily living tasks such as putting a cup of teaon a desk. Coping with IT is therefore a critical issue for the CNS and adaptively using it may contribute to motor efficiency by lowering the overall amount of muscle torque (MT) required to achieve a given task. In contrast, its inadequate use or inefficient exploitation may amplify sensorimotor noise throughout the kinematic chain or lead to greater energy expenditure. Interestingly, a large body of evidence has suggested that the CNS has ability to estimate and anticipate IT via internal models of the limbs’ dynamics (e.g. ref. 1) (but see ref. 2 for an alternative view based on equilibrium-point theory). In this study, we will rely on the internal model hypothesis and hypothesize that the CNS has some form of knowledge about IT at the motor planning stage. In this view, we may devise two extreme options but the solution used by the brain can conceivably lie in-between. On the one hand, the “compensation” hypothesis assumes that IT is totally canceled itself out during motor planning^3^,^4^,^5^,^6^,^7^,^8^,^9^. On the other hand, the “exploitation” hypothesis assumes the brain utilizes IT as much as possible to generate motion at other DoFs without dedicated muscle effort at those DoFs^10^,^11^,^12^,^13^,^14^,^15^,^16^,^17^,^18^. Arguably, the extent to which the CNS actually exploits IT should be reflected in the adaptability of arm trajectories with respect to speed and load changes as IT strongly depends on such factors. Hence the purpose of this study was twofold: (i) evaluate whether the CNS adaptively exploits (or compensates) IT during unrestrained 3D arm movements, and (ii) identify normative principles that may account for how the CNS copes with IT during motor planning.

To this aim, we investigated a free 3D arm pointing task and modeled it using optimal control theory. The rationale of our approach was as follows. First, to let emerge motor strategies revealing IT exploitation or compensation strategies, subjects were asked to perform movement without a prescribed reach endpoint. Precisely, we considered unconstrained 3D arm pointing movements to a horizontal planar target. This laboratory task can be thought as similar to pushing a door to open it or to the cup of tea example given above; in these tasks, there is no unique final hand position for their achievement^19^,^20^. The present task nonetheless imposes accuracy constraints forcing the participants to spatially control their end-effector along the vertical direction in order to reach the goal (i.e. the plane here). Therefore, participants had to take into account IT during planning or execution to perform the task successfully just as in classical point-to-point reaching paradigms. Considering 3D motion was also critical since it fundamentally differs from 2D motion in that IT does not only exist between segments but also within a segment in 3D. Second, to better characterize the role of IT, we varied both the inertia (addition of a load to the forearm) and the speed (slow/fast verbal instructions) as the shape and magnitude of IT critically depends on those factors. We predicted that, if IT is exploited (even partially), the inter/within-segment ITs should contribute more significantly to trajectory and torque profile formation when inertia and/or motion speed are large. Alternatively, if IT is compensated, such modifications should have non-significant effect on the arm trajectories, except that the MTs should be adjusted to cancel the larger ITs. Third, we aimed at accounting for the empirical pointing strategies via inverse optimal control with composite cost functions^21^,^22^,^23^. Optimal control theory is an appealing framework to apprehend human motion as it points out possible high-level principles underlying arm movement formation in a concise and normative way^24^,^25^,^26^. Here, kinematic costs are necessarily associated with a “compensation” strategy because they ignore IT during motor planning while energetic and dynamic costs may fall within the “exploitation” category as they take into account IT at the planning stage. To the best of our knowledge, it is the first time that the role of IT on 3D human motion is interpreted from the (inverse) optimal control viewpoint. We hypothesized that any adaptive compensation/exploitation trade-off of IT could be associated to the minimization of composite cost mixing kinematic, dynamic or energetic quantities.

## Results

### Kinematic analysis

#### Hand kinematics

**Figure 1:**
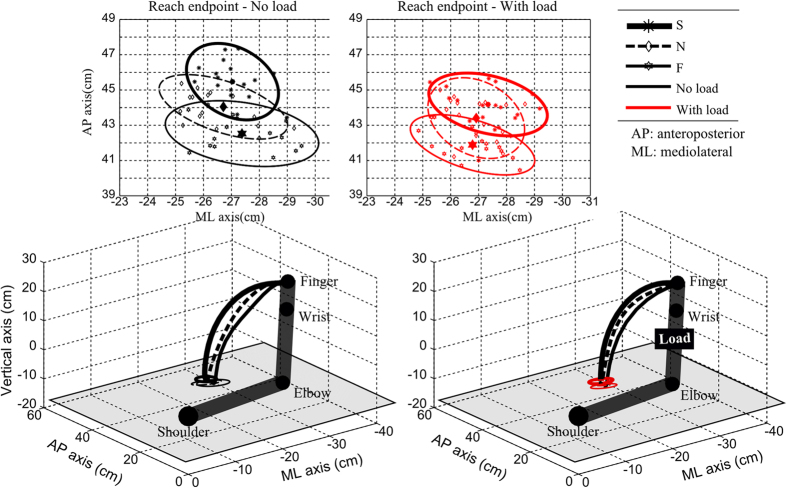
Illustration of the experimental paradigm. Fixed initial arm position and horizontal target plane were tested, therefore defining a free reach-endpoint motor task. A 4-DoF model of arm was examined (3 DoFs at the shoulder and 1 DoF at the elbow). Three speed and two load conditions were tested. At the *two bottom panels* displayed the arm posture at the initial time with no load (*left*) and with a load (*right*) approximately attached to the center of mass of the forearm. The average fingertip trajectories of a representative subject were drawn in thick, thin and dotted lines for the three speeds (slow, natural, fast, denoted by S, N, F) respectively. The two *top panels* display the reach endpoint positions across trials for this subject for the three speed condition, and no-load *(left*) and load (*right*) conditions. The 95% confidence ellipses of the reach endpoints are drawn. Note that along the antero-posterior (AP axis), the position of reach endpoint positions tended to get closer to the shoulder position when movement speed increased or when the load was attached to the forearm.

**Figure 2:**
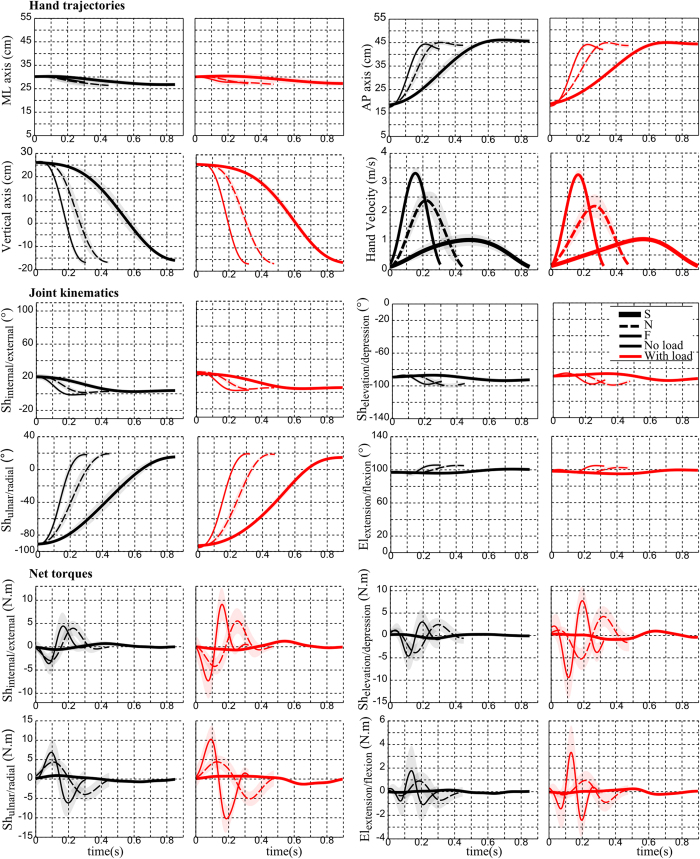
Hand, joint and torque profiles for the representative subject of Fig. 1. For the hand kinematics, displacements along the AP, ML and vertical axes are depicted as well as the Cartesian hand velocity (average and standard deviation shown as a shaded area) for the 3 speeds and two load conditions (black is for no-load and red for with-load). For the joint kinematics, the angular displacements for the 4 degrees of freedom are depicted. For the joint torques, we depicted the net torque acting at each degree of freedom.

The task is illustrated in Fig. 1 for a representative participant where the initial posture, hand paths, final positions and the planar target are represented. The same participant will be illustrated throughout the paper to facilitate qualitative comparisons between figures. The complete hand kinematics is depicted in Fig. 2 (top panel) for this representative participant. Overall, classical patterns seen in point-to-point reaching movements were observed. The movement was mainly along the AP and vertical directions, with typical sigmoidal patterns, and the velocity was bell-shaped for all speeds. Although the reach endpoint was not imposed in this free-endpoint task, we thus observed quite classical hand kinematic patterns. A quantitative analysis using data from all the participants is given hereafter.

##### Movement duration (MD)

Movement durations for the two experimental blocks (i.e. no-load and with-load), averaged across all subjects, are presented in Table 1. For the former block, the values (mean ± std) corresponding to the three speed conditions (i.e. S, N, F) were respectively 0.85 ± 0.15, 0.49 ± 0.07, 0.32 ± 0.06 s. Those values were respectively 0.84 ± 0.14, 0.52 ± 0.08, 0.35 ± 0.07 s for the latter block. Two-way repeated measures ANOVAs showed no speed × load interaction for MD (p = 0.55). However, significant differences were found across speed conditions (p < 0.001, F(2, 18) = 101.1, 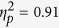), but not between load conditions (p = 0.13). This result, therefore, implied that the speed-related requirements (i.e. distinguishable within each load condition but consistent between two load conditions) were fulfilled by all subjects during the experiment.

##### Finger endpoint variability (Z_a_ and Z_p_)

The finger constant and variable errors along the vertical axis (*Za* for accuracy and *Zp* for precision) were averaged across all subjects and displayed in Table 1. It is noteworthy that the pointing accuracy along the horizontal axes (AP and ML) were not examined because no prescribed reach endpoint was introduced regarding to the final position of the fingertip on the horizontal plane. Therefore there was no constant error in this plane. Regarding the goal of the task (thus along the vertical axis), *Zp* values slightly increased while *Za* values slightly decreased as movement speed increased, regardless of the load condition. However, two-way repeated measures ANOVAs analyses showed no significant load × speed interaction or load/speed variation for *Za* error (*p*_*load×xspeed*_ = 0.13, *p*_*load*_ = 0.83, *p*_*speed*_ = 0.46) and for *Zp* error (*p*_*load×speed*_ = 0.37, *p*_*load*_ = 0.44, *p*_*speed*_ = 0.08), thus indicating equal accuracy/precision achievement despite different speed/load movement conditions. In order to evaluate whether the change of speed/load conditions affected the shape of arm trajectories, the following analyses focuses on other relevant and classical kinematic parameters.

##### Reach endpoint analyses

One main variable of the task is the reach endpoint location as it was left free to the participants. We first qualitatively examined the effect of speed and load variations on the reach endpoint position. To this aim, the projection of reach endpoints onto the transversal plane (composed of ML and AP axes) is presented in the top panels of Fig. 1 for the representative subject. A visual inspection suggests that the distribution of reach endpoints (displayed as 95% confidence ellipses) varies with respect to the different speed and load conditions. Specifically, the subject executed movement trajectories whose endpoint got closer to the shoulder’s vertical projection along the AP axis when movement sped up. A similar shift of the reach endpoint toward the shoulder location was observed when adding a load to the forearm. Similar analyses were systematically conducted for all subjects. The normalized reach endpoint index (*RE*_*AP*_, see Materials and Methods for its definition), averaged across all subjects is displayed in Table 1. Results showed that the reach endpoint index systematically decreased when movement speed increased and when the load was attached to the arm. This index was [76.3 ± 7.9%, 74.2 ± 9.3%, 73.6 ± 9.3%] for the no-load condition and [74.4 ± 8.2%, 72.0 ± 8.9%, 71.3 ± 9.6%] for the with-load condition, respectively. Two-way repeated measures ANOVAs showed no significant speed × load interaction (p = 0.83) but a significant change of *RE*_*AP*_ with respect to speed (p < 0.001, F(2, 18) = 12.2, 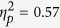) and load (p < 0.05, F(1, 9) = 5.2, 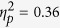) changes. This analysis proved that the final hand position, which was freely chosen in this task, changed as a function of speed and load across subjects: subjects tended to point closer to their body when speed or forearm inertia increased.

##### Curvature index analysis

The averaged curvature indexes are reported in Table 1 for the load and speed conditions. For the no-load condition, the index obtained values of 0.18 ± 0.03, 0.17 ± 0.03, 0.16 ± 0.03 for the S, N and F speeds respectively. These values were equal to 0.18 ± 0.03, 0.17 ± 0.03, 0.16 ± 0.04 respectively for the load condition. Two-way repeated measures ANOVAs revealed no significant speed × load interaction (p = 0.07) and no significant effect of load (p = 0.78) but a significant change of curvature index under speed variations (p < 0.001, F(2, 18) = 13.3, 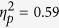). Therefore, hand paths tended to become straighter with speed increments.

#### Joint kinematics

Like the hand kinematics, the joint kinematics was also illustrated for the representative subject in Fig. 2 (middle panel). It is visible that the movements were executed mainly around the shoulder ulnar/radial axis while smaller displacements were observed for the other remaining axes. The magnitude of these angular displacements were analyzed for all subjects and the results are displayed in Table 1. Two-way repeated measures ANOVAs showed no significant speed × load interaction and no significant effect of load and speed for shoulder internal/external, elevation/depression, elbow extension/flexion but a significant change for the shoulder ulnar/radial angle under speed variations (p < 0.001, F(2, 18) = 27.8, 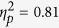). The extent of the rotations around this major axis for the task depended on speed but not load.

In summary, the speed and load dependencies of the reach endpoint and curvature indexes suggested that the CNS may adapt its motor control strategy to best suit the new task constraints. In order to better uncover the possible causes underlying this adaptation, an analysis at the dynamic level is performed hereafter.

### Dynamic analysis

#### Qualitative analysis of torque profiles

For the sake of illustration, net torque (NT) profiles are shown in Fig. 1 for all conditions of speed and load (bottom panels) for the representative subject. The four torque profiles corresponding to the internal/external, elevation/depression, ulnar/radial DoFs of the shoulder and the extension/flexion DoF of the elbow are depicted. Overall, net torque profiles marked clear differences with respect to load or speed variations. The magnitude of net torques typically increased when the load was added or when speed was augmented by a verbal instruction. The patterns were similar to that of an acceleration in most cases, except for the elbow extension/flexion in fast speed. In terms of magnitude, the torques at the elbow joint appeared to be considerably smaller than those produced at the shoulder joint. Within the shoulder joint itself, the torque profiles of the three coupled DoFs were quite complex. Although kinematic analyses showed less involvement of shoulder internal/external and elevation/depression axes in the formation of arm trajectories compared to the shoulder ulnar/radial axis (Table 1), one could however observe quite large torques at the two former axes. Maximum magnitude was about 10 N.m and measured at fast speed (F).

**Figure 3:**
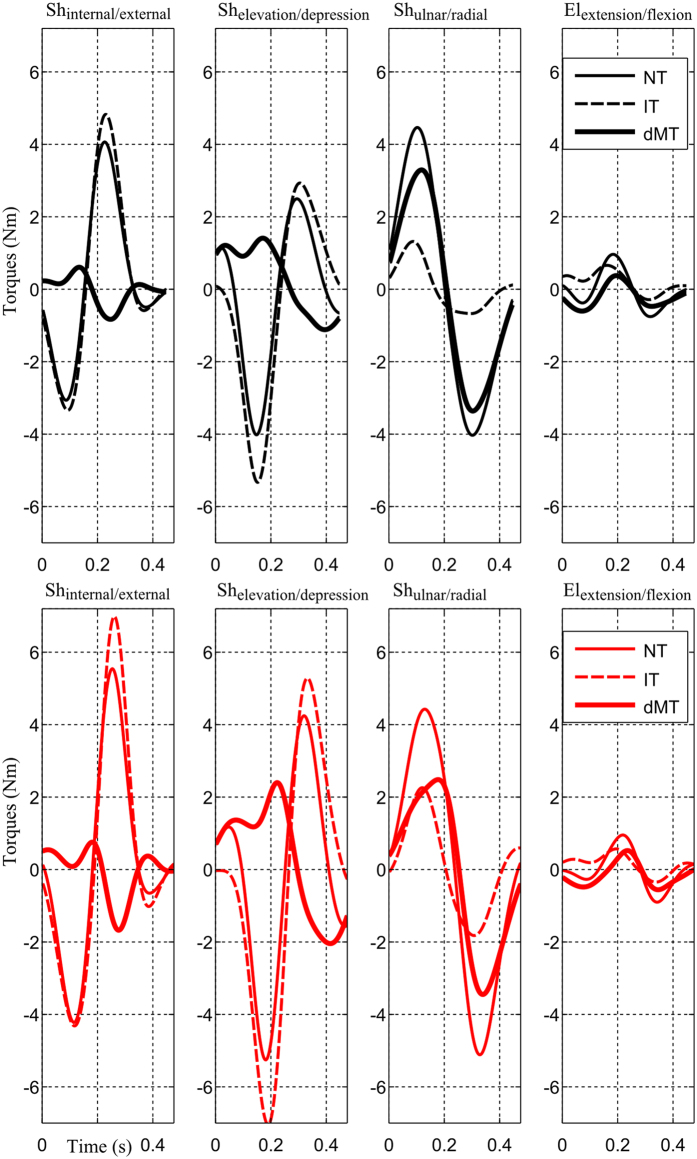
Torque profiles (averaged across trials) of the representative subject at N speed for the no-load (*top panel)* and load *(bottom panel)* condition*s.* From left to right, the four torque profiles are for the shoulder internal/external, elevation/depression, ulnar/radial and elbow extension/flexion DoFs, respectively. The dynamic muscle torque (defined as muscle torque deprived of gravity torque, denoted by dMT), interaction torque (denoted by IT) and net torque (denoted by NT) are plotted.

For a more detailed analysis of the composition of net torques, a focus on normal speed (N) is presented in Fig. 3 in the two experimental load conditions (no-load/with-load) and for the same representative subject. Other speeds are not illustrated here for clarity but the effect of speed will be treated in the subsequent quantitative and statistical analyses. Specifically, at the prominent axis for the task (i.e. shoulder ulnar/radial), the dynamic muscle torque (defined as muscle torque without the gravity component, denoted as dMT) and the interaction torque (IT) both contributed to the net torque (NT) in the same direction, thus producing quite large NT here. On the contrary, for the other axes (e.g. shoulder internal/external and elevation/depression), the dMT was most of the time opposed to both IT and NT; meanwhile, IT was observed to vary in the same direction with the NT.

A finer examination however revealed few factors that may be responsible for the speed/load-dependent arm kinematics found above. Indeed, in terms of magnitude, while the maximal value of NT at the shoulder ulnar/radial axis in the load condition was pretty similar to that of the no-load condition (4 Nm), the difference in the NT for the two load conditions was much larger at the shoulder internal/external and elevation/depression axes. Interestingly, it is noteworthy that the motion of the internal/external and elevation/depression DoFs of the shoulder joint should have high impacts on the location of the final reach endpoint within the planar target (and maybe curvature too). Therefore, these differences on mean torque profiles, especially at the first two shoulder rotation axes, could possibly account for the speed/load-dependent changes of reach endpoint/curvature observed above.

Interestingly, note that the magnitude of IT increased clearly with the addition of the load (as expected) and that it reached quite large compared to the other torques (dMT, NT). Moreover, IT appeared to vary systematically in the same direction than NT (i.e. assisting the motion), thereby explaining what dMT were relatively small in general. Therefore, from these plots, it seems possible that the CNS may take into account the presence of IT to facilitate and assist movement. In order to clarify this hypothesis, we next quantitatively examined the impact of IT on NT in terms of *IT*^*g*^ and *IT*^*l*^ indexes (see Materials and Methods for their definitions), which are indexes designed to assess the degree of contribution of IT to NT.

#### Torque profile quantitative analyses

##### Global interaction torque index (*ITg*)

**Figure 4:**
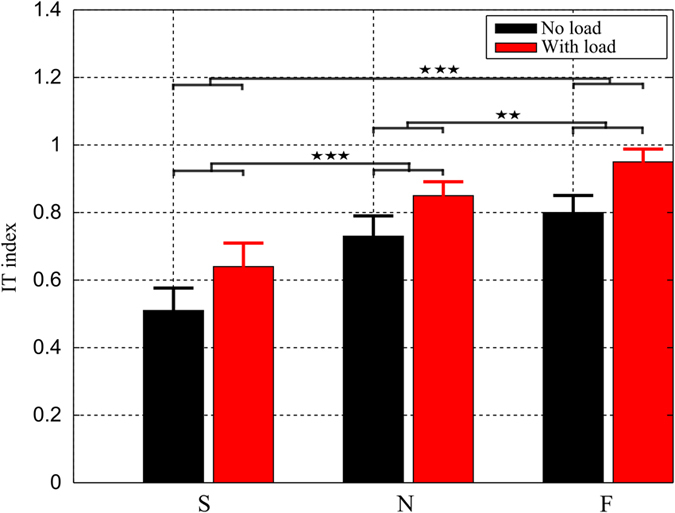
Global interaction torque indexes (*IT*^*g*^), averaged across all subjects, and displayed for the three speed and two load conditions (with standard errors indicated by error bars). It is visible that the *IT*^*g*^ index increased whenever movements sped up or a load was attached to the arm. In addition, its values were always positive, thus indicating that the IT positively contributed to the NT to some extent. Note that horizontal bars with stars indicate the results of post-hoc analysis for the speed condition. One, two, three stars stand for p < 0.05, p < 0.01 and p < 0.001 respectively.

The *IT*^*g*^ indexes, averaged across all subjects for both speed/load experimental conditions, are shown in Fig. 4. In terms of load effect, it is visible that the *IT*^*g*^ indexes were larger in the load condition than in the no-load condition, regardless of motion speed. Precisely, the *IT*^*g*^ indexes shifted from [0.51 ± 0.21, 0.73 ± 0.19, 0.80 ± 0.16] for the no-load condition to [0.64 ± 0.22, 0.85 ± 0.13, 0.95 ± 0.12] for the load condition with respect to S, N, F speeds, respectively. In terms of speed effect, the *IT*^*g*^ indexes of both load conditions increased whenever the movement was sped up. Two-way repeated measures ANOVAs on *IT*^*g*^ yielded no significant speed × load interaction (p = 0.56) but significant effects of load (p < 0.001, F(1, 9) = 89.1, 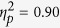) and speed (p < 0.001, F(2, 18) = 41.2, 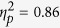), indicating that IT were exploited to a greater extent whenever the load and the movement speed were increased. Moreover, it is noteworthy that *IT*^*g*^ values were always positive (>0.5) while they could theoretically be negative as well. Altogether, these results implied that in general IT assisted the movement and that, importantly, this contribution of IT to the movement was strengthened with load or speed augmentations (which increase overall IT magnitudes).

#### Interaction torque contribution characteristics

##### Spatial investigation: local interaction torque indexes (*IT^l^*)

**Figure 5:**
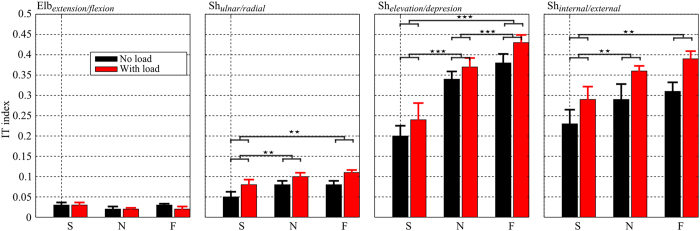
Local interaction torque indexes (*IT*^*l*^), averaged across all the subjects, displayed for the three speed and two load conditions. From left to right: *IT*^*l*^ of elbow extension/flexion, shoulder ulnar/radial, elevation/depression, internal/external, respectively. Noticeably, compared with shoulder-related DoFs, the *IT*^*l*^ indexes at the elbow extension/flexion were considerably smaller. Between the three DoFs at the shoulder, the *IT*^*l*^ indexes of shoulder ulnar/radial were smaller than the others. Statistical analyses showed significant effects of speed/load on the *IT*^*l*^ index for these three DoFs, as indicated by horizontal bars

In order to better understand the mechanism the CNS may rely on to adaptively use IT at different DoFs, we investigated the local IT contribution index (*IT*^*l*^) for every rotation axis involved in the limb’s movement. The averaged *IT*^*l*^ indexes for both load and speed conditions are given in Fig. 5. Like *IT*^*g*^ index, we observed that the *IT*^*l*^ index was also speed and load dependent. Indeed, for most DoFs (except the elbow extension/flexion axis), two-way repeated measures ANOVAs on *IT*^*l*^ indexes showed no significant speed × load interaction but a significant increase of *IT*^*l*^ (*p*_*uln*/*rad*_ < 0.01, *F*_*uln*/*rad*_ (1, 9) = 19.2, 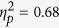; *p*_*ele*/*dep*_ < 0.001, *F*_*ele*/*dep*_ (1, 9) = 20.2, 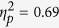; *p*_*int*/*ext*_ < 0.001, *F*_*int*/*ext*_ (1, 9) = 41.4, 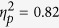) when the load was attached to the arm or when the movement sped up (*p*_*uln*/*rad*_ < 0.001, *F*_*uln*/*rad*_ (2, 18) = 11.6, 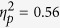; *p*_*ele*/*dep*_ < 0.001, *F*_*ele*/*dep*_ (2, 18) = 30.7, 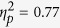; *p*_*int*/*ext*_ < 0.001, *F*_*int*/*ext*_ (2, 18) = 14.9, 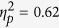). Finer analysis showed that IT was utilized to assist movement at several DoFs but to different extents. Indeed, for the two load conditions, the IT indexes of elbow extension/flexion axis were always smaller than those of shoulder-related ones. Between the three coupled rotation axes of the shoulder joint, the IT indexes of shoulder internal/external and elevation/depression axes were considerably larger than those of shoulder ulnar/radial axis irrespective of speed/load conditions. Given that these three axes belong to a same joint, these results revealed the complexity of IT contribution.

##### Temporal investigation: interaction torque index analysis across bins

**Figure 6:**
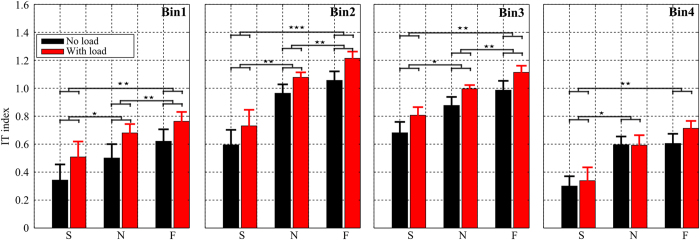
Bin analysis of interaction torque exploitation. From left to right: average interaction torque indexes (across subjects) for bin 1, bin 2, bin 3 and bin4 respectively. Note that these four bins were defined by dividing movement duration into a series of 4 intervals based on the acceleration profile (see Methods). Note that IT index values were smaller for bins 1 and 4, while it was larger for middle bins (2 and 3). For each bin, statistical significance of post-hoc tests is reported.

The binned global IT indexes, averaged across all subjects, are illustrated in Fig. 6. It is visible that the pattern of IT indexes over the four bins for the two load conditions remains relatively consistent across all movement speed conditions. At normal speed (N) for example, the IT indexes of four bins clearly changed in a manner that is reminiscent of the bell shape of the hand velocity profile. Indeed, when the subjects started or terminated motion (bin 1 or bin 4), the IT index obtained the relatively small values of [0.50 ± 0.31, 0.59 ± 0.18] for the no-load condition and of [0.68 ± 0.20, 0.59 ± 0.22] for the load condition. Around the peak of velocity (i.e. bin 2 or bin 3), the IT index increased considerably and reached [0.96 ± 0.2, 0.87 ± 0.19] for the no-load and [1.07 ± 0.11, 0.99 ± 0.08] for the load conditions respectively. This may not be a surprise given that IT magnitude depends on speed and accelerations, which are greater during the course of a reaching movement than near the initiation or termination phases. A main effect of speed on IT index was also present for each of the four bins. Two-way repeated measures ANOVAs on IT index showed no significant speed × load interaction but a significant effect of speed for all bins (*p*_*bin*1_ < 0.001, *F*_*bin*1_(2, 18) = 13.1, 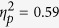; *p*_*bin*2_ < 0.001, *F*_*bin*2_(2, 18) = 20.1, 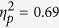; *p*_*bin*3_ < 0.001, *F*_*bin*3_(2, 18) = 12.2, 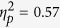 and *p*_*bin*4_ < 0.001, *F*_*bin*4_(2, 18) = 11.1, 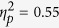). In terms of load effect, the binned IT indexes were generally consistent with the above results. Indeed, the IT indexes in the load condition were always significantly larger than those in the no-load condition for the first three bins (*p*_*bin*1_ < 0.001, *F*_*bin*1_(1, 9) = 39.6, 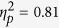; *p*_*bin*2_ < 0.01, *F*_*bin*2_(1, 9) = 18.2, 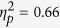; *p*_*bin*3_ < 0.01, *F*_*bin*3_(1, 9) = 13.6, 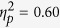) but no significant difference was found for the fourth bin (*p*_*bin*4_ = 0.13).

##### Nature of interaction torque: velocity- versus acceleration-based IT index

**Figure 7:**
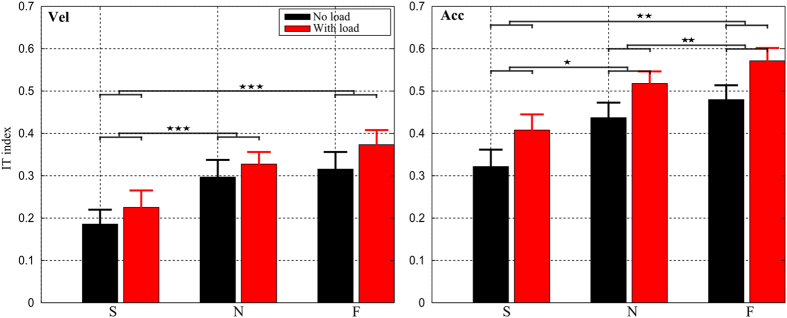
Component analysis of interaction torque exploitation. The IT indexes, averaged across subjects, are displayed for the three speed and two load conditions for the two components of IT. *Left*: velocity-related component (*IT*_*vel*_); *Right*: acceleration-related component (*IT*_*acc*_). It is visible that *IT*_*acc*_ was always greater than *IT*_*vel*_. Statistical analyses showed significant effects of speed and load on the IT index for both IT components.

The averaged IT indexes across all subjects taken separately for the two interaction torque components (*T*_*acc*_ and *T*_*vel*_) are reported in Fig. 7 (see the Materials and Methods section for their definitions). Overall, the IT indexes of the two torque components were quite similar, suggesting that IT originating from velocities and accelerations are both used during the movement. Indeed, both IT indexes increased whenever movement speed increased. In terms of load effect, we also observed an upward shift of IT index for both *T*_*acc*_ and *T*_*vel*_. Two-way repeated measures ANOVAs showed no significant speed × load interaction (*p*_*Tacc*_ = 0.85, *p*_*Tvel*_ = 0.48) but significant main effects of speed/load conditions for both *T*_*acc*_ (*p*_*speed*_ < 0.001, *F*_*speed*_(2, 18) = 14.9, 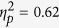; *p*_*load*_ < 0.001, *F*_*load*_(1, 9) = 95.4, 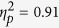) and *T*_*vel*_ (*p*_*speed*_ < 0.001, *F*_*speed*_(2, 18) = 34.3, 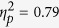; *p*_*load*_ < 0.001, *F*_*load*_ (1, 9) = 21.9, 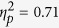). Finer analysis revealed that the IT indexes of *T*_*acc*_ were always larger than those of *T*_*vel*_ regardless of speed/load conditions but their difference remained approximately constant with respect to the change of speed/load. Nonetheless, it is remarkable that the IT indexes of both *T*_*acc*_ and *T*_*vel*_ were always positive, thereby implying that in all cases both *T*_*acc*_ and *T*_*vel*_ contributed positively to NT and that, overall, IT assisted the movement.

In summary, the above empirical results revealed a significant effect of speed and load on both the kinematics and dynamics of the motor behaviors during the free 3D arm pointing movements we considered here. This effect seems to originate from the unequal influence of IT on NT when speed or/and load are modified. Next, to interpret and propose principles to explain the above kinematic and kinetic adaption of reach strategies to load and speed variations, inverse optimal control simulation results are presented. Inverse optimal control is dedicated to automatically characterize the cost with respect to which empirical trajectories are assumed to be optimal. In other words, this is done to characterize the best-fitting composite cost for the experimental motion data under investigation.

### Optimal control results

#### Arm kinematics predicted by composite and elementary costs

**Figure 8:**
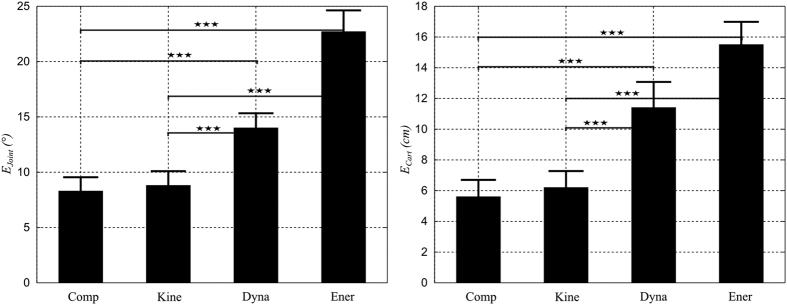
Reconstruction errors in joint space (*E*_*Joint*_, *left panel*) and Cartesian space (*E*_*Cart*_, *right panel*) for the best-fitting composite cost and each of the three cost elements taken separately. Error values were averaged across speeds, loads and then across subjects (with standard errors indicated by error bars). Noticeably, in terms of both joint and Cartesian errors, the composite (Comp) and kinematic (Kine) costs performed better than the dynamic (Dyna) and energetic (Ener) costs.

We applied direct optimal control (DOC) separately to the three elementary cost functions under consideration (see Table 2) for all subjects to check whether each of them could account for the experimental observations. These simulated optimal trajectories were used to compute errors in terms of angular displacements (*E*_*Joint*_) and Cartesian displacements (*E*_*Cart*_), which were then used as reference values for comparisons with the performance of the composite cost obtained from the inverse optimal control (IOC) procedure, as illustrated in Fig. 8. The Cartesian error *E*_*Cart*_ of the composite and kinematic costs were equal to 5.5 ± 3.2 cm and 6.2 ± 3.4 cm respectively. These errors were clearly smaller than those of the dynamic cost (*E*_*Cart*_ = 11.4 ± 5.3 cm) and even much smaller than those of the energetic cost (*E*_*Cart*_ = 15.5 ± 4.7 cm). Repeated measures one-way ANOVAs showed significant differences between the composite/kinematic costs and the dynamic/energetic costs (p < 0.001, F(3, 177) = 79.6, 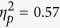) but no significant difference between the kinematic and composite cost (p = 1.0) when pooling all experimental conditions together. Similar conclusions were reached for the joint space errors (*E*_*Joint*_). Like the Cartesian errors, the angular errors (*E*_*Joint*_) of the composite and kinematic costs were quite small compared to those of the dynamic/energetic costs. Repeated measures one-way ANOVAs for *E*_*Joint*_ errors also showed significant differences between the composite/kinematic costs and the dynamic/energetic costs (p < 0.001, F(3, 177) = 125.8, 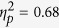) but the difference between the kinematic and composite cost was not significant (p = 1.0). Altogether, one can conclude that both composite and kinematic costs performed better than the dynamic/energetic costs at replicating the main kinematic features of the recorded data. Therefore, pure energetic and dynamic costs can be ruled out as they are clearly unable to predict accurately some basic features of the task (e.g. hand path), and the composite and kinematic costs appear as the only candidates to account for the experimental trajectories at this point. In order to distinguish between these two costs, we further examined their performances in dynamic space. In particular, we investigated whether the IT index and its speed/load dependencies were replicated by such models.

#### Arm dynamics predicted by composite and kinematic costs

**Figure 9:**
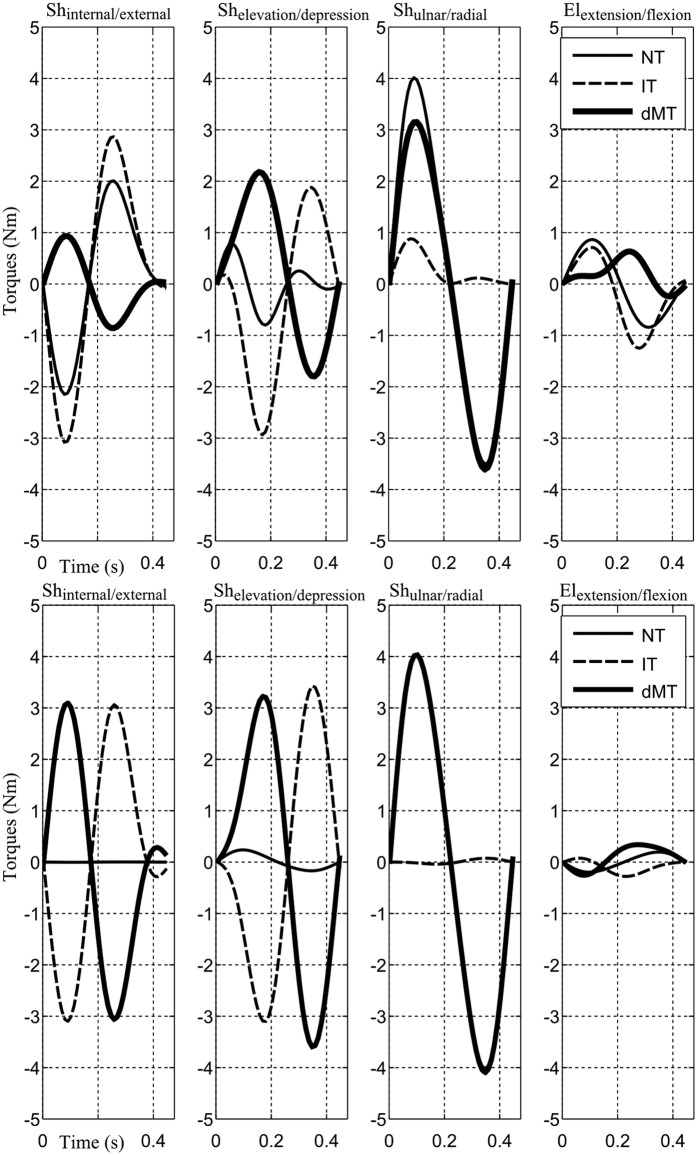
Torque profiles predicted by the identified composite cost (*top panels*) and the kinematic cost (*bottom panels*) for the representative subject at N speed and in no load condition. It is visible that the composite cost tends to let ITs contribute to NTs in order to get smaller dMTs while it is the opposite for the kinematic cost.

For the sake of illustration, the dynamic strategies predicted by the identified composite and kinematic costs at normal speed (N) are shown in Fig. 9 in the no-load condition for the representative subject. It is visible that the two costs predicted quite different toque profiles if one considers the interplay between torques. Indeed, for the kinematic cost, large magnitudes of dMTs were required mainly to cancel out the perturbations caused by ITs, especially at the first two DoFs (i.e. shoulder internal/external and shoulder elevation/depression, about 3 Nm). Moreover, it happened that IT was opposed to NT, especially for the 2^nd^ DoF. On the other hand, for the composite cost, smaller dMTs were required to perform the task and, clearly, this was achieved by letting IT contribute to NT on certain time periods. To assess the consistency of this observation, we further quantified their performance in terms of IT index as presented below.

**Figure 10:**
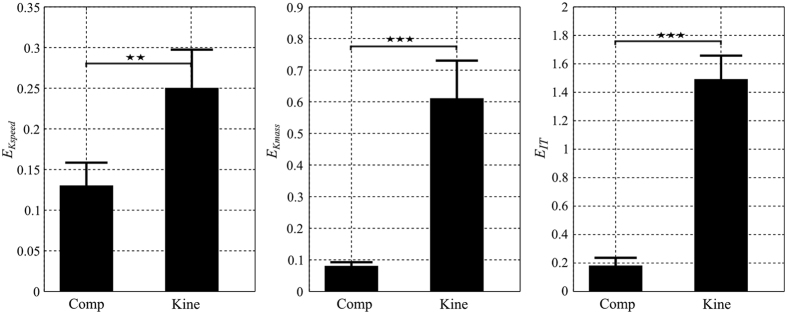
Reconstruction errors for some relevant parameters (*E*_*IT*_, *E*_*Kspeed*_, *E*_*Kmass*_) for the composite and kinematic costs. Error values were averaged across speeds, loads and then across subjects for *E*_*IT*_ while averaged only across subjects for *E*_*Kspeed*_/*E*_*Kmass*_. Noticeably, in terms of *E*_*IT*_ and *E*_*Kspeed*_/*E*_*Kmass*_ errors, the composite cost performed much better than the kinematic cost, yielding to a conclusion that only composite cost could predict relatively well the IT index and its speed/mass dependencies as observed in the experimental movement.

We first calculated the absolute reconstruction error of IT index (*E*_*IT*_) for both composite and kinematic costs. Their *E*_*IT*_ value was averaged across speeds, loads and then across subjects and displayed in the right panel of Fig. 10. Importantly, the composite cost predicted the IT index much better than the kinematic costs. Indeed, the (mean ± std) *E*_*IT*_ values of the composite cost were equal to 0.18 ± 0.16, nearly eight times smaller than those of the kinematic cost (*E*_*IT*_ = 1.49 ± 0.53). Given that the empirical IT indexes (*IT*^*g*^) always obtained positive values larger than 0.5, the predicted IT index of the kinematic cost was considerably discrepant from the recorded data. Indeed, the kinematic cost exhibited such large *E*_*IT*_ errors because it often produced trajectories whose IT indexes were negative (opposite to the real ones) irrespective of speed/load conditions. Repeated measures one-way ANOVAs for *E*_*IT*_ showed significant difference between the composite cost and the kinematic cost (p < 0.001, F(1, 59) = 420, 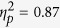).

Furthermore, we tested if the composite cost better reproduced the speed/load dependencies of IT indexes than the kinematic cost. To this aim, the prediction of slope obtained from a linear regression of IT index against speed/load variables was investigated. The left and middle panels of Fig. 10 show the errors 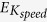 and 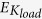. In terms of speed effect, it is visible that the optimization of a composite cost predicted the speed-dependent change of IT indexes better than the kinematic cost. Indeed, the 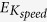 error was 0.13 ± 0.09 for the composite cost, which was nearly twice smaller than for the kinematic cost (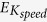 = 0.25 ± 0.15). Repeated measures one-way ANOVAs for 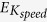 showed significant difference between the composite and kinematic costs (p < 0.01, F(1, 19) = 8.8, 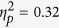). In terms of load effect, the difference between the composite and kinematic cost was even larger. Compared with the composite cost, the kinematic cost replicated quite poorly load-dependent variations (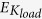 = 0.61 ± 0.38, i.e. nearly seven times larger than the error for the composite cost). Again, repeated measures one-way ANOVAs for 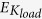 revealed that the composite and kinematic costs differed significantly (p < 0.001, F(1, 9) = 20.7, 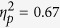). Overall, our results therefore revealed that the composite cost could capture reasonably well both the adaptive kinematic and dynamic characteristics of the reach strategies while the kinematic cost clearly failed to explain the reach strategies in torque space.

#### Cost contribution evaluation

**Figure 11:**
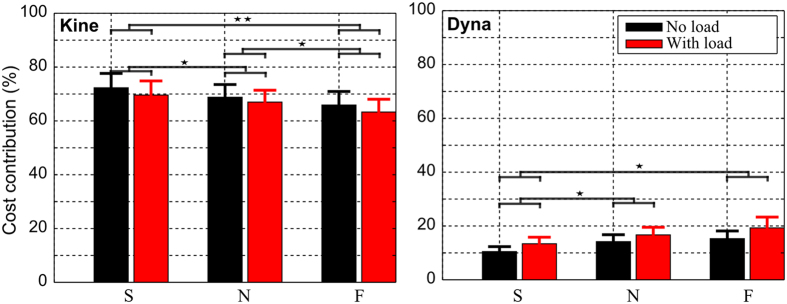
Cost contribution analyses. The contribution of elementary cost to the composite cost, averaged across all the subjects, is reported for the three speed and two load conditions. *Left*: cost contribution of kinematic cost; *Right*: cost contribution of dynamic cost. It is visible that the contribution of kinematic cost tended to decreased while those of dynamic cost increased with respect to the increase of movement speed and load.

In order to assess how each elementary cost contributed to the total (composite) cost according to speed and load changes, we estimated the influence of each of the kinematic, dynamic and energetic components (see Materials and Methods) and the results are reported in Fig. 11. Here, we display only results of the kinematic and dynamic costs since the three cost contributions sum to 100% by definition. Overall, the contribution of the kinematic cost was largely dominant. Specifically, the average contribution (across speed and load conditions and subjects) of the kinematic cost was about 67 ± 14% while those of energetic and dynamic elements were 16 ± 11% and 17 ± 18% respectively. This result thus confirmed the important role played by the kinematic cost during the motor planning process, which is consistent with its role for replicating the kinematics of the arm pointing movements.

Further analyses showed that the contribution of each cost with respect to the composite cost varied whenever movements sped up or a load was attached to the forearm (even if the combination weights were fixed). Particularly, the kinematic contribution tended to decrease while the energetic and dynamic contributions increased. Indeed, when the speed increased from slow to natural and then to fast, the kinematic contribution reduced on average by [4%, 7%] while the energetic and dynamic contribution increased amount of [0.5%, 1%] and [3.5%, 6%] respectively. Two-way repeated measures ANOVAs yielded no significant speed × load interaction (*p*_*kine*_ = 0.73; *p*_*ener*_ = 0.09; *p*_*dyna*_ = 0.39) but a significant effect of load (*p*_*kine*_ < 0.05, *F*_*kine*_(1, 9) = 5.9, 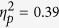; *p*_*ener*_ < 0.05, *F*_*ener*_(1, 9) = 6.8, 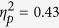; *p*_*dyna*_ < 0.01, *F*_*dyna*_(1, 9) = 13.9, 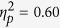) and speed (*p*_*kine*_ < 0.001, *F*_*kine*_(2, 18) = 12.6, 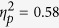; *p*_*ener*_ < 0.05, *F*_*ener*_(2, 18) = 2.9, 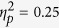; *p*_*dyna*_ < 0.01, *F*_*dyna*_(2, 18) = 9.3, 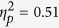) on the contribution values for all variables.

In summary, these results show that the CNS may largely rely upon a kinematic cost to plan motion kinematics but that dynamic/energetic cost elements are crucial to account for the kinetics of the reach strategies and their adaptation to speed and load variations. This interpretation is confirmed by a reduction of the kinematic cost contribution which is gradually replaced by a larger contribution of its dynamic and energetic counterparts as speed and/or load increase.

## Discussion

In the current work, we examined the extent to which the brain adaptively exploits or compensates the interaction torque (IT) to assist or resist human arm movements and analyzed where this could originate from. To this aim, a free 3D arm pointing task (without predefined reach endpoint) was investigated while varying both limb inertia and movement speed, two factors that are known to influence IT. The experimental results showed that IT partly contributed to net torque (NT) thereby assisting the movement and that such contribution increased with limb inertia and movement speed. This finding might either be due to a lack of explicit compensation for IT or be a purposive goal of the CNS in order to exploit IT whenever it is sensible. This question was tackled by assuming that the observed trajectories were optimal with respect to a certain optimality criterion, and results showed that the present empirical observations were overall compatible with a composite cost trading-off kinematic, energetic and dynamic variables and not by any of these costs taken individually. Moreover, the increment of IT-to-NT contribution index was associated with a decreased contribution of the kinematic cost to the composite cost. This may shed new light on the origin of the adaptive use of IT, which might be related to the optimization of a trade-off between motion smoothness (i.e. kinematic) and effort (i.e.energetic/dynamic) that inherently reshapes the kinematic and kinetic aspects of a movement depending on speed/load constraints. These results are discussed in details hereafter.

### Load and speed dependent use of interaction torque

Both load and speed was varied in this study in order to clarify the role of IT in motion planning. Indeed, IT critically depends on limb’s inertia and speed characteristics. So if IT is an integral part of motion planning, some relevant motion parameters should vary significantly with respect to load/speed variations during the considered free pointing task. In the literature, several studies varied loads to examine the extent to which the brain tunes motor planning according to inertial properties. This was done in a series of works which have shown that motion kinematic parameters such as movement paths, endpoint variability and normalized velocity profiles were load-independent^27^,^28^,^29^,^30^,^31^. These findings thus argued for a load compensation strategy since no effect of load on the arm trajectories was observed. However, this compensation strategy was questioned by other studies^32^,^33^,^34^ showing that the kinaesthetic perception of limb’s position was significantly affected by rotational inertia variables such as the minimum inertia principal axis. Therefore, the brain seems to take into account not only the load but also the specific distribution of the masses involved in rotational movements and might therefore use it during motor control. This latter idea found support in recent studies which showed significant effects of the racket polar moment of inertia on the limb movement strategy when examining tennis serve^35^,^36^. However, these studies were either limited to the analysis of the kinematic aspect of motor tasks or only concentrated on analyzing the effect of load on muscle torque (MT) while the effect of load on IT was not thoroughly examined, especially regarding to the contribution of IT to other torques (e.g. NT or MT). In the present work, using a quantitative approach to estimate the contribution of IT to NT allowed us to establish a more direct link between the limb inertia and the selected motor command defining the muscle patterns driving the arm. Interestingly, our results revealed that the brain purposely let IT increasingly contribute to NT to assist the movement when the limb inertia was increased via the addition of a load on the forearm.

Similar to the load effect, our results showed a significant effect of speed regarding the role of IT in the control of free arm pointing. Quantitative analyses showed that IT contributed more to NT such that IT assisted the movement more when the movement sped up. This finding was interesting given that IT magnitude is also known to increase drastically with speed, which was already shown to lead to IT utilization strategies in throwing tasks^11^,^12^,^15^,^16^,^17^,^37^. Further examination of IT at different phases of the movement (Fig. 6) showed that the contribution of IT to the movement was the highest around the peak of velocity. This finding was coherent with the work of^18^ where the effect of IT was proved to vary with different temporal phases of the movement during a catching task. Additionally, the IT indexes related to accelerations (*T*_*acc*_) and velocities (*T*_*vel*_) both increased with respect to the movement speed. This finding actually confirms the work of^3^ which showed that the IT velocity terms have the same order of magnitude than the IT acceleration terms for a large range of movement speeds.

We next addressed the question of what principles or rules account for such an adaptive speed/load dependent use of IT. Namely, is it due to a lack of explicit compensation for IT or a goal purposely planned by the CNS in order to exploit IT? In the subsequent paragraph, we discuss why the brain could both neglect and utilize IT depending on the time instant and DoF, and why discriminating between compensation and exploitation of IT is sometimes a challenging task.

### Compensation or exploitation of interaction torques, or both?

In the literature, the role of IT (being compensated or exploited) has long been debated and our study may help to disentangle some controversial interpretations. The compensation of IT has been proposed to account for the invariant aspects of certain movement kinematic parameters such as straight hand paths and bell-shaped speed profiles and extensively investigated in several studies of multi-joint limb movement focusing on planar point-to-point reaching tasks^2^,^3^,^4^,^5^,^6^,^7^. These studies argued that the explicit compensation of IT could remove the non-linearity and noise-related errors caused by IT in control signals, thereby contributing to stabilize and smooth out the movement. This hypothesis is actually compatible with the kinematic-based motor planning principles such as the well-known minimum jerk^27^ as it allows using a simple “scaling law” to accommodate various movement speeds^3^. The common aspect of these studies is that the experimental setup was designed for planar motion involving quite a small number of DoFs (usually 2), and usually constrained the arm motion (e.g. via a manipulandum) and imposed a specific predefined reference point as a target, which may have affected their control strategies^38^. In some studies, the setup could even lead subjects to freeze themselves the motion at specific DoFs. This was the case in the work of^4^ where the same elbow excursion was always required whereas shoulder excursion could vary, or by the work of^7^ where the experimental setup allowed accomplishing the task by only rotating around 1 DoF while keeping the motion state at another DoF either stationary or unchanged. As such, these protocols might have induced an IT compensation strategy in the sense that it was somewhat necessary to guarantee the task requirements. Nonetheless, it demonstrated clearly that the brain has the ability to estimate and accurately compensate for IT when relevant for the task.

On the other hand, when examining more complex movement tasks (usually involving more than 3 DoFs) and without predefined final configuration of the limb (e.g. overthrowing task^11^,^12^,^15^,^16^,^39^), some authors have rather argued for the exploitation of IT to assist the movement. Interestingly, this latter idea allowed to explain many experimental observations. For instance^39^, showed that the inability to exploit the passive inter-segmental interaction forces was associated with the poor ability to throw fast balls in cerebellar and unskilled subject. In a cyclical arm rotation task^40^, showed that different rotation axes were chosen by different subjects, which was interpreted as different levels of IT exploitation depending on individual sensorimotor characteristics (more visual or proprioceptive). These findings were actually generalized into the hierarchical control hypothesis^11^,^12^ or the leading joint hypothesis^14^ that stressed the role of shoulder as a fundamental motion generator at other joints, via inter-segmental interaction acting at distal joints. Common to all these studies was the optimization of performance, which was a clear task objective (e.g. throw a ball at maximal speed will require a subject to effectively coordinate torques to gain more acceleration), and the freedom offered by the motor task (relatively weak spatial constraints).

The above discussion emphasizes a possible link between the role of IT and the requirements of the task. It suggests that (i) the brain can compensate or exploit IT depending on the characteristics of the task and (ii) compensation or exploitation may not be a mere property of the motor controller; instead, it could be the consequences of higher processes within the brain that are related to the subjective and objective goals of the movement. Therefore, if the compensation versus exploitation debate seems to have a task-dependent origin, difficulties also arose because the answer could only be about a “partial exploitation” or “partial compensation” in general. Indeed, what is not full compensation can be seen as partial exploitation and vice-versa. In most existing studies which supported the compensation of IT^2^,^3^,^4^,^5^,^6^,^7^, subjects failed to perfectly counterbalance the effect of IT. It was then argued that this was due to the lack of explicit compensation of IT, or to a partial compensation of IT^7^. In fact, there was still a small contribution of IT to NT. Similarly, in studies supporting IT exploitation, IT was never observed to contribute completely to NT, meaning that a part of IT was actually canceled out by MT. In other words, the joints (often distal) were not entirely moved by IT, likely because such perfect exploitation would have driven the system to states incompatible with the task achievement or have yielded to undesired arm kinematics (e.g. more jerky trajectories). Therefore, it seems tricky to conclude whether the brain plans to partially compensate or partially exploit IT, but what is undeniable is that the brain elaborates motor commands such that IT contributes to NT whenever it is possible and relevant for the task. We discuss below how this relative exploitation versus compensation strategies may arise during motor planning.

### Motor planning: a trade-off between kinematic and kinetic factors

The above issue about IT compensation/exploitation is in fact strongly related to the nature of hypothetical cost functions underlying motor planning. At a theoretical level, it is clear that the optimization of a kinematic cost would produce maximally smooth movements that account for the case of a full compensation of IT. In contrast, the optimization of dynamic or energetic costs would minimize the magnitude or the work of MT which will result in IT exploitation, to the greatest possible extent. In other words, this will improve the efficiency of the motor controller with respect to motion effort. Therefore, the question of whether the brain exploits or compensates IT during movement planning can be re-approached using optimal control theory and comparing between kinematic versus dynamic or energetic cost functions. Indeed, kinematic cost functions cannot exploit IT while cost functions involving torque-related variables can exploit IT. In this vein, assessing whether human movement is planned in terms of kinematic or dynamic/energetic variables has been tested in several studies^41^,^42^,^43^,^44^,^45^,^46^. Here, optimizing only energetic or dynamic criteria lead to discrepant arm trajectories in terms of joint and Cartesian displacements, in which case it made no sense to further investigate what happened in torque space. In contrast, maximizing smoothness in joint space (angel jerk, i.e. kinematic cost) was remarkably efficient to fit the angular and Cartesian displacements but it failed to describe accurately the movement in torque space. The only model that could explain both kinematic and kinetic aspects of the reach strategies, and their speed/load dependences, was the composite optimality criterion mixing variables of different nature. This composite cost idea was already advanced and investigated differently in previous studies^21^,^22^,^23^.

Interestingly, the effectiveness of a composite cost mixing these criteria to replicate the experimental data may help to understand why the reach strategies planned by the brain were as observed, in particular for what concerns ITs. For instance, one could wonder why the brain did not let IT drive the movement more extensively (e.g. at the 1^st^ and 2^nd^ DoF of the shoulder joint). Indeed, less MT would be required at these DoFs, which may be dynamically more efficient. However, in this case the arm trajectory would have been very different from the empirical one, possibly quite jerky, and the existence of large IT might even be harmful for the anatomical arm structure. One could also wonder why the brain did not try to cancel out all ITs to gain movement smoothness and stability but also to simplify motor planning. In that case, the brain should totally compensate or even “overcompensate” for ITs, that is, having both NT and MT opposed to IT at a given DoF. However, the big disadvantage would be that large muscle torques are required to do so. Therefore, although motor planning could be simplified by neglecting IT effects and subsequently canceling them out during motor execution, the dynamic efficiency of the movement would simply be non-optimal. Reconciling all the advantages and disadvantages of these two extreme strategies, the empirical and simulation results indicated that the brain may choose an mixed motor planning principle combining both kinematic and kinetic variables whose relative contributions to the control strategy may be differentially revealed by the task demands. This composite cost may automatically yield the adaptive IT compensation/exploitation trade-offs described in the present study.

### Limitations

At this point, it worth stressing some limitations of the present work. First, the superiority of the composite cost over the kinematic cost requires 2 additional parameters for each participant. Therefore, it is questionable whether the improvement in the fitting is large enough to justify these additional tuning parameters. To clarify this point, we computed Akaike Information Criterion (AIC) according to the formula *AIC* = *n* log(*RSS*/*n*) + *k* where *n* is the number of samples, *RSS* is the residual sum of squares for the predicted variable and *k* is the number of parameters plus one^47^. An optimal control model predicts a whole movement and, therefore, many parameters can be extracted and used to compare simulated and experimental trajectories. Here we used a kinematic measure (RE index) and a dynamic measure (IT index) to assess the two models. AIC values for the kinematic and composite cost models were respectively 27.0 ± 3.9 and 26.6 ± 3.1 for RE index. This means that there was no obvious need for a composite cost function to account for the experimental hand paths. At this level of analysis, using a composite cost was possibly related to overfitting. When looking at the predictions in dynamic space (via IT index this time), AIC values were 6.7 ± 1.0 and −15.2 ± 2.5 for the kinematic and composite costs respectively. At this level of analysis, using a composite cost with adjustable parameters was therefore well justified. Here we relied on two important parameters of the present tasks, however optimal control models predict plenty of parameters. These considerations emphasize a key question in motor control: what metric or parameters should be used to compare simulated and experimental data in general? While kinematics should likely be first predicted before dynamics, it is conceivable that a good model should explain motion in both spaces at once.

Second, one should also stress that our formalism might overestimate the role of the kinematic cost in motor planning. Indeed, we used deterministic optimal control in which a feedforward motor plan is completely established before movement is executed. This complete planning of the trajectory is not strictly required by the optimal feedback control (OFC) formalism that assumes a feedback control law whose characteristics moreover depend on the signal-dependent noise present in the nervous system^48^. In a similar task with redundant targets, we have shown in an earlier study that OFC was especially useful to account for inter-trial variability via the minimum intervention principle^19^ (which should explain ellipses in Fig. 1). However, when looking at average behaviors, a deterministic modelling was overall consistent with such models^22^. Signal-dependent noise may nevertheless reshape the mean optimal trajectories to some extent. This could affect the kinematic model that requires large MT in this deterministic setting. With signal-dependent noise and a variance cost on the endpoint it is conceivable that such large MT would be penalized as they would increase motor variability. But variability increases with “effort” as noise is multiplicative, which is related to the size of motor command (which could be related to the torque change in our simple model). Therefore, a kinematic and variance cost would be reminiscent of the composite cost investigated in the present deterministic framework.

## Materials and Methods

Ten right-handed subjects (5 men) agreed to participate in the study. All of them were healthy and ignorant of the goal of the scientific work. Age, weight and height (mean std) were 27 ± 5 years, 67 ± 10 kg and 168 ± 7.8 cm, respectively. All subjects were made aware of the protocol and written inform consents were obtained before the study. Experimental protocol and procedures were approved by the Univ. Paris-Sud EA 4532 local Ethics Committee and carried out according to the ethical guidelines conforming to the Revised Helsinki Declaration of 2000.

### Experimental task

In the present study, we used a “manifold reaching” paradigm quite similar to the experiment described in the work of ref. 23. Briefly, participants sat comfortably on a chair and were asked to perform pointing movements with their right (dominant) upper-limb to an horizontal planar target (Fig. 1). Participants were asked to stop their motion when their index fingertip was onto the target but without striking it. They started from an initial “L-shape” arm configuration where the elbow was approximately flexed at 90 degrees with an upper arm abducted to the horizontal and aligned with the mediolateral direction; this starting posture was realized in practice by asking the participants to put their fingertip at a reference point whose position was adjustable in space. In this way, the initial arm joint configuration was kept similar across participants. Free rotations at the shoulder and elbow joints were allowed while the wrist joint was constrained by a light bar to freeze its motion and simplify subsequent modeling. The trunk was fixed and attached to the chair. The planar target consisted of an horizontal foam-made surface positioned just below the participant’s chest on a table (about 17.5 cm below the shoulder on average). Participants could reach anywhere on the surface without moving the shoulder and the maximal distance they could reach to was limited by their full arm’s length and shoulder’s height with respect to the planar target. The size of the foam-made surface was 40 cm in the ML axis and 65 cm in the AP axis and placed to the right hemibody. During movement execution, participants were asked to look at a reference point placed on the wall in front of them (in the direction of Frankfurt plane). Importantly, no specific instruction about the reach endpoint location was given in this task and the participants were free to choose their preferred final hand position as long as it lay on the planar target. As such, the task was redundant because an infinity of reach endpoints were compatible with task achievement as well as an infinity of final joint configurations. Here the goal of the task was to control the fingertip error along the vertical axis and it mostly involved four degrees of freedom (DoF) of the arm: three at the shoulder joint and one at the elbow joint (the elbow pronation/supination was neglected because of its minor role in a pointing task). Therefore, the set of permissible joint configurations lay on a 3-D manifold (4 joint angles minus 1 equation for the plane constraint).

For the purpose of this study, three speed conditions and two load conditions were investigated. We used a block design for the load. The three speeds, slow, natural and fast and denoted by S, N and F respectively, were randomized across trials to prevent habituation and memorization effects when subjects switched from one speed to another. Load conditions were counterbalanced across subjects. At the beginning of each trial, participants were verbally instructed about the imposed speed by the experimenter and no feedback was given about their actual speed except if it was clearly erroneous. We first recorded a block of 45 trials without load attached to the participant’s forearm (no-load condition, 15 trials per speed). In the second block of 45 trials, we attached a load approximately to the center of mass of the forearm (load condition). The load was a thick-walled cylindrical tube whose inner/outer radius was adjustable with respect to the subject’s forearm size. The tube’s mass was set equal to 0.4, 0.6 or 0.8 kg, depending on the weight of the subjects whose values were smaller than 56 kg, between 56–65 kg or larger than 65 kg, respectively. These individual settings were introduced to adjust the arm dynamics modification to the participant’s actual weight. Before the experimenter started to record, participants trained for 30 trials familiarize with the pointing task (both no- and with- load conditions). During this process, their performance was visually checked by the experimenter who made sure that the initial arm postures were consistent across trials, that the gaze was directed towards the requested location during movement execution, that speed differences were clearly marked (i.e. S, N and F) and, finally, that subjects stopped their one-shot movements accurately enough on the planar target. Note that adaptation to a load has been previously shown to be very fast in reaching studies (a couple of trials^49^ and hence these training trials were assumed to be enough for the participants to reach a stable level of performance before the true experimental recordings began. In summary, for each participant, 15 trials were tested per experimental condition (no-load/load, S/N/F speeds), such that 90 trials were recorded and analyzed per participant. In total, 900 trials were therefore analyzed in this study.

### Data Recording

The motion kinematics was recorded at a frequency of 500 Hz by using a 8-camera optical motion capture system (Vicon Inc. Oxford, UK). A total of 13 plug-in-gait markers were attached to specific anatomical locations on the dominant arm and other parts of the body as follows: seventh cervical vertebrae, 10^th^ thoracic vertebrae, clavicle, sternum, right and left acromion, lateral and medial humeral epicondyles, ulnar and radial styloids, 2^nd^ and 5^th^ metacarpal heads and 1^st^ finger tip. Noise was filtered out from the recorded positions of the markers by using a 2^nd^-order Butterworth low-pass filter at 10 Hz. These 3D positions were then analyzed at kinematic and dynamic levels using a custom-written software in Matlab (Mathworks, Natick, MA), as detailed in the sequel.

### Kinematic analysis

#### Hand kinematics

For each trial, the 3D positions of the fingertip marker were numerically differentiated to obtain the endpoint velocity profile. Based on this velocity profile, the movement duration was estimated as the largest time interval where the velocity is above 5% of its maximal value. Other hand kinematic parameters relevant to the purpose of the present study (e.g. reach endpoint, curvature, vertical accuracy/precision errors) were then calculated, as follows:

- *Index of path curvature* (*Cur*). The index *Cur* was computed as *Cur* = *D*_*max*_/*D*_*isp*_ where *Dmax* is the maximal deviation of the finger from the straight line connecting the initial to the final finger position during the movement and *Disp* is the length of the latter Euclidean distance.
- *Reach endpoint* (*RE*). The *RE* was defined as the 3D coordinates of the fingertip position at the end of the reach, namely the anteroposterior (AP, X axis), mediolateral (ML, Y axis) and vertical (Vert, Z axis) coordinates relative to a frame centered at the shoulder joint. In the present study, the Z-coordinate of the RE was used to quantify accuracy and precision of the pointing movement (as the target plane was defined by an equation Z = constant). In contrast, the X- and Y- coordinates of the fingertip were freely chosen by the participants, and therefore, were used to assess the reaching strategy selected by the subjects and not pointing errors. When relevant, a normalized *RE* position along the AP axis (*RE*_*AP*_) was used in the analyses. It was defined as the ratio between the AP coordinate of the *RE* and its maximum possible value (attained when the subject fully extended the arm without moving the trunk).
- *Vertical accuracy/precision error calculations* (*Za* and *Zp* indexes). The constant error (*Za*) was defined as the averaged distance from the final fingertip position to its vertical projection on the planar target, while the variable error (*Zp*) was defined as the standard deviation of reach endpoint positions in the vertical direction across trials, within one experimental condition. The examination of *Za* and *Zp* allowed verifying the extent to which subjects stopped their movements in the vicinity of the target plane as required.

#### Joint kinematics

The joint kinematics (e.g. angular displacements, velocities and accelerations) was estimated based on a method previously described in refs 23 and 40. Local coordinate systems was built from the markers in agreement with the guidelines of the international society of biomechanics (ISB). From the relative orientation of the coordinate systems, rotation matrices and then Euler angles (namely internal/external, elevation/depression, ulnar/radial at shoulder and extension/flexion at elbow) were calculated. At last, the joint speeds and accelerations were evaluated via numerical differentiations.

### Dynamic analysis

In order to examine the effect of load and speed on the dynamics of the reaching behavior, a dynamic model of the arm was introduced to allow estimating the muscle, net, interaction and gravity torques. The dynamic model used has also been described in prior works (e.g. ref. 23).

As it is usual, the arm dynamics was described by the following equation:


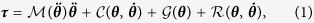


where *τ* denotes the muscle torque, 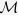 the inertia matrix (4 × 4 here), 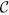 the Coriolis/centripetal torque, and 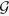 the gravity torque vector. The term 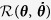 reflects the residual torques created by soft tissues, which affect muscle torque in practice. Here we assumed that 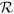 was small compared to the other torques and we thus ignored it in the present modeling. The vector ***θ*** = (*θ*_1_, …*θ*_4_)^Τ^ describes the arm’s configuration and time derivatives are indicated with a dot (or multiple dots) throughout the paper.

For the purpose of this article, let us define the following quantities:


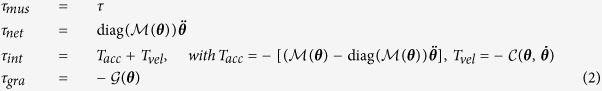


where 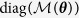 is the diagonal matrix built from the diagonal terms of the mass matrix. Then, equation 1 can be compactly written as


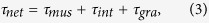


which is similar to the description given in refs 50 and 51.

To simulate this dynamics in practice, we employed the Featherstone-Newton-Euler algorithm, which is the state-of-the-art of rigid body algorithms^52^. Having efficient algorithms is crucial, not for performing inverse dynamics and recovering experimental torque profiles, but for running inverse and direct optimal control simulations as they involve numerous evaluations of the system dynamics.

### Direct and Inverse optimal control

We conducted direct and inverse optimal control investigations along the lines of refs 22 and 23. Briefly, this approach involves the definition of a cost function that usually depends on the set of control variables (denoted here by **u**, chosen to be the derivative of joint accelerations, i.e. jerk) and the set of controlled variables describing the state of the musculoskeletal system (such as joint angles, velocities and accelerations, denoted by **x**). As such, the definition of the considered optimal control problems is as follows.

#### Direct optimal control (DOC)

The direct optimal control problem corresponding to the cost *C*(**u**, x) can be formalized as follows: *Find the optimal control* **u** *and its associated trajectory* **x** *satisfying the dynamical system equation, connecting an initial resting arm posture to a terminal one such that the endpoint lies on the planar target in time T and yielding a minimal value of the cost C*(**u**, x).

We examined three cost functions representing the three main classes of existing costs (i.e. kinematic-oriented, energy-oriented and dynamic-oriented respectively, see Table 2). Namely, we considered the integral of squared angle jerk (kinematic cost), the work of absolute values of muscle torques (energetic cost) and the integral of squared torque change (dynamic cost). This selection was based on the fact that these three costs were all found relevant in previous arm reaching studies^53^,^54^,^55^,^56^,^57^. We could also have included other optimality criteria based on angle acceleration or muscle torque but the acceleration cost yielded the same joint path as the angle jerk and the muscle torque predicted irrelevant hand path trajectory for the current task (see ref. 22 for more details). For the sake of simplicity and clarity, we thus assumed that the cost function accounting for the experimental trajectories could be a composition of the three elementary costs defined above, as follows:


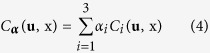


where *C*_*i*_(**u**, x) (*i* = [1, 2, 3]) are defined precisely in Table 2. The vector ***α*** = (*α*_*i*_)_1≤*i*≤3_ is referred to as the tuning vector whose elements verify *α*_*i*_ ≥ 0. The process of searching a best-fitting composite cost is called inverse optimal control and is summarized below (more details can be found in refs 22, 23 and 58). Note that only two parameters were actually free in the above IOC problem due to normalization (the solution of *C*_***α***_(**u**, x) is the same than *λC*_***a***_(**u**, x) for any positive λ).

#### Inverse optimal control (IOC)

The inverse optimal control problem is stated here as a ≪bi-level≫ optimization. The lower level simply solves a DOC problem for a given vector ***a*** and serves to obtain optimal simulated arm trajectories 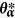. The higher level aims at finding the optimal vector 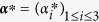 that yields the minimal value of an error function comparing the experimental trajectories ***θ***^*meas*^ with the optimal simulated ones. This function was denoted by Φ and was defined as 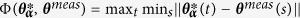. It captures the maximum deviation from the simulated joint displacements (which depend on the components of ***α***) to the experimental path in joint space. We will refer to this as *E*_*Joint*_, which can be expressed in degrees.

In practice, we used the Matlab-based software called GPOPS to solve the DOC (lower level). For the higher level and IOC, a derivative-free method called CONDOR was used. For both DOC/IOC processes, the initial/final angle velocities and angle accelerations were set to zeros while the initial arm posture (i.e. four initial examined angles) and the movement duration (*T*) were imported from the motion capture data.

#### Cost contribution (in %)

We used the formula 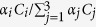 to evaluate the contribution of cost *i* to the total cost^22^ (costs are evaluated for the optimal solution). Looking at cost contributions may be useful because elements of the vector ***a*** are not always easy to interpret: the cost associated with the largest *α*_*i*_ might play a minor role on trajectory formation depending on the order of magnitude of the other elements of the composite cost. Note that from this definition, the 3 cost contributions sum to 100%.

### Interaction torque analysis

#### Local and global interaction torque indexes

Formulas to assess the contribution (either positive/negative) of IT to NT have been established in previous works but they were mainly designed for 2-DoF planar arms^10^,^50^. Here, the 4-DoF arm model required some modifications and extensions of those formulas. A drawback of previous approaches was that they were designed for individual DoFs, making it difficult to get a concise picture of the overall interaction torque contribution for such a system with more DoFs. Here we wanted to compare the 4 interaction torques and evaluate their involvement in the formation of reach strategies. Therefore, we defined two complementary indexes. The first one, called “*local IT index*” and referred to as 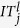 allowed analyzing the contribution of IT to NT at each DoF relatively to the others wherein the subscript *j* refers to the four DoFs under consideration. The second one, called “*global IT index*” and referred to as *IT*^*g*^, allowed revealing the overall contribution of IT to NT. Precisely, the formulas were as follows:


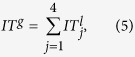



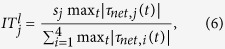



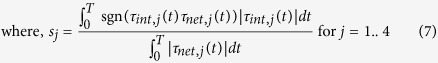


where sgn is the standard signum function.

The rationale was twofold: (i) contribution of IT to the movement at each DoF was assumed to be revealed by the amount of time during which IT contributed to the corresponding NT modulated by the absolute magnitude of this IT and normalized by the magnitude of the NT and (ii) the role of IT at a prominent DoF for the movement under consideration is more important than at other DoFs. In practice, we first adopted the equation proposed by^10^,^50^ to calculate a sub-index (denoted as *s*_*j*_, for DoF *j*), which is given in Eq 7. These resulting sub-indexes were then combined with the maximal value of NT at each DoF to yield the local IT index for DoF *j* (i.e. 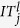) as illustrated in Eq 6. By taking into account the magnitude of the corresponding NT, the 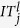 index allowed comparing the contribution of IT to NT between different DoFs. The global interaction torque index (i.e. *IT*^*g*^) was then defined as a sum of the local *IT*^*l*^ indexes of all 4 DoFs involved into movement as illustrated in Eq 5. The resultant value reflected the extent to which the IT overall acted with the NT to assist movements (i.e. globally supporting the motion if the obtained *IT*^*g*^ value was positive) or against the NT to resist movements (i.e. globally opposing the motion if the obtained *IT*^*g*^ value was negative).

#### Binned interaction torque indexes

In the previous formula, the contribution of IT to NT was computed from the whole movement duration (from the movement onset *t*_0_ to the movement end *t*_*f*_). However, because IT depends on speed, these contribution indexes could also depend on the movement phase. In order to evaluate this, we split the whole time window into four consecutive intervals (i.e. 4 bins) and quantified the IT index for each bin separately (using *IT*^*g*^ formula restricted to that bin). Precisely, these four bins were defined from the acceleration profile of finger tip as follows: the 1st interval was specified by the time period starting from *t*_0_ to the instant when the fingertip acceleration obtained its maximal value (denoted by *t*_max_); the 2nd bin was defined from *t*_max_ to the time when the fingertip acceleration canceled itself out (crossing the horizontal axis; denoted by *t*_zer_); the 3rd interval was estimated from *t*_zer_ to the instant when the fingertip acceleration reached its minimal value (denoted as *t*_max_); finally, the 4th interval was the remaining part of the movement, measured from *t*_max_ to the end of the motion *t*_*f*_.

#### *T*_*acc*_ versus *T*_*vel*_ examination

As noticeable from Eq. 2, IT is actually composed of two different components (*T*_*acc*_ and *T*_*vel*_) whose formulas are based on angular acceleration and velocity variables, respectively. Therefore, it is interesting to assess whether the motor controller exploited separately these two components to assist or resist the movement. To this aim, we relied upon *IT*^*g*^ formula but restricted IT to either its velocity or acceleration component. Precisely, in Eq. 7, we replaced the total IT torque *τ*_*int*_ by its elements (*T*_*acc*_ and *T*_*vel*_) to get the respective *s*_*j*_ values before applying Eqs 5 and 6 to obtain the desired indexes.

#### Comparison between simulated and experimental movement strategies

##### Cartesian error (*E*_*Cart*_)

In order to evaluate the simulated arm trajectories in Cartesian space, the 3D trajectory of the fingertip was analyzed. More precisely, the Cartesian error was estimated as the maximal deviation from the simulated finger trajectory to its experimental path and denoted by *E*_*Cart*_ (this is the Cartesian analog of *E*_*Joint*_).

##### Absolute IT index error (*E_IT_*)

In order to evaluate the extent to which the simulated trajectories accounted for IT indexes, the absolute IT index error was computed. It was denoted by *E*_*IT*_ and defined as the mean absolute difference between simulated and experimental IT indexes across all speed and load conditions (*IT*^*g*^). It was computed for each subject separately.

##### Relative IT index error: load- and speed-related errors ( 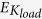 and 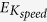 )

To assess the extent to which the simulated results could reproduce the load and speed variations of IT index, we compared empirical and simulated slopes of linear regressions of IT indexes (*IT*^*g*^) against the load and speed. For the load condition, the slope *K*_*load*_ was computed based on a linear regression of *IT*^*g*^ values against the load variable whose values were taken equal to zero and the real weight of attached load with respect to the no-load/load conditions for every subject. For the speed condition, the slope *K*_*speed*_ was calculated separately for each load condition and resulted from linear regressions of *IT*^*g*^ values against the speed variable whose values (corresponding to S, N, F speeds) were imported directly from the recorded data (simply taken as the maximal value of recorded velocity profile) for each subject.

### Statistical analyses

Two-way repeated-measures ANOVAs were used to test the effects of load and speed on certain pertinent movement parameters. Moreover, in order to assess the predictive performance of each cost function with respect to the others, one-way repeated-measures ANOVAs were also used when relevant. Post-hoc analyses were conducted with Bonferroni corrections when relevant and a 5% threshold was selected in all cases to reject the null hypotheses. Shapiro-Wilk statistics was used to evaluate normality for the parameters under investigation. We used SPSS to perform all statistical analyses.

## Additional Information

**How to cite this article**: Vu, V.H. *et al*. Adaptive use of interaction torque during arm reaching movement from the optimal control viewpoint. *Sci. Rep.* **6**, 38845; doi: 10.1038/srep38845 (2016).

**Publisher's note:** Springer Nature remains neutral with regard to jurisdictional claims in published maps and institutional affiliations.

## Figures and Tables

**Table 1:** Main kinematic movement parameters (mean ± std across subjects) for the three speed (S, N, F) and two load (no load, with load) conditions.

	No load			With load		
	S	N	F	S	N	F
MD (s)	0.85 ± 0.15	0.49 ± 0.07	0.32 ± 0.06	0.84 ± 0.14	0.52 ± 0.08	0.35 ± 0.07
*Sh*_*internal*/*external*_ (°)	11.3 ± 6.6	12.5 ± 6.3	14.1 ± 6.3	11.2 ± 5.5	12.7 ± 5.8	12.2 ± 6.2
*Sh*_*elevation*/*depression*_ (°)	10.3 ± 7.0	11.4 ± 6.3	11.8 ± 6.5	9.9 ± 5.3	10.6 ± 5.2	11.2 ± 5.2
*Sh*_*ulnar*/*radial*_ (°)	96.4 ± 16.1	98.8 ± 15.9	100.5 ± 16.1	97.0 ± 15.1	99.5 ± 14.9	101.7 ± 16.1
*El*_*extension*/*flexion*_ (°)	14.7 ± 8.6	14.6 ± 7.6	14.7 ± 6.9	13.1 ± 8.6	12.4 ± 8.7	12.9 ± 8.2
*RE*_*AP*_ (%)	76.3 ± 7.9	74.2 ± 9.3	73.6 ± 9.3	74.4 ± 8.2	72.0 ± 8.9	71.3 ± 9.6
Cur	0.18 ± 0.03	0.17 ± 0.03	0.16 ± 0.03	0.18 ± 0.03	0.17 ± 0.03	0.16 ± 0.04
*Za* (cm)	0.8 ± 0.7	0.7 ± 0.7	0.6 ± 0.4	0.7 ± 0.6	0.6 ± 0.6	0.6 ± 0.5
*Zp* (cm)	0.7 ± 0.2	0.8 ± 0.3	0.9 ± 0.5	0.7 ± 0.2	0.8 ± 0.3	0.8 ± 0.3

**Table 2:** Definition and references for the cost functions used in the current study.

Criterion	Cost function	References
Kinematic (*Angle jerk*)	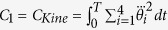	53
Energetic (*Absolute work*)	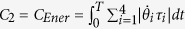	54,55
Dynamic (*Torque change*)	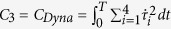	56,57
